# Prevalence and Correlated Factors for Obesity, Overweight and Central Obesity in Southwest of Iran

**Published:** 2019-07

**Authors:** Seyed Bahman GHADERIAN, Leila YAZDANPANAH, Hajieh SHAHBAZIAN, Ali Reza SATTARI, Seyed Mahmuod LATIFI, Sara SARVANDIAN

**Affiliations:** Diabetes Research Center, Health Research Institute, Ahvaz Jundishapur University of Medical Sciences, Ahvaz, Iran

**Keywords:** Obesity, Overweight, Central obesity, Body mass index, Prevalence

## Abstract

**Background:**

We assessed the prevalence of obesity, overweight, central obesity and their associated risk factors in an urban population in Ahvaz, southwest of Iran.

**Methods:**

This population-based cross-sectional study was performed via random cluster sampling method in 6 health centers in Iran in 2015. A questionnaire was completed by each individual.

**Results:**

Of 2575 participants, 1187 (46%) were men. Nearly 50% of the participants’ level of education was high school or higher. About 82% of the population was married and about one-third had positive family history of parental obesity. The total prevalence of obesity, overweight, and central obesity were 26.5%, 38.7%, and 28.6%, respectively. The rate of obesity in men was lower than in women (*P*<0.001). The prevalence of obesity increased until the age of 60 yr in both genders and decreased thereafter except for central obesity in women, which increased without any lag. Low level of education, marriage, positive history of parental obesity and parity ≥five were associated with increased odds of obesity (OR=2.14(1.52, 3.00), OR=2.4(1.75, 2.99), OR=2.7(1.71, 3.2) and 4.16(2.17, 7.65), respectively).

**Conclusion:**

Obesity and overweight have a high prevalence in southwest of the country, increasing with age. Although several risk factors are associated with obesity, the prevalence of obesity and overweight can be reduced by controlling the risk factors in the community.

## Introduction

Obesity is an unfavorable consequence of changes in lifestyle and behavioral patterns. It is also associated with many chronic diseases including diabetes, hyperlipidemia, cardiovascular disease and osteoarthritis (1–3). Obesity might result in early disability and consequential unwanted retirees in a vast number of patients due to osteoarthritis as well as diabetes and cardiovascular-associated complications. Medical cost of obesity-related morbidities is estimated to be about 147 billion dollars per year in the USA. Therefore, it has imposed a huge economic burden on societies (4).

The role of both nurture and nature in development of obesity are undisputed since the contribution of genetic susceptibility may account for about 30%–70% of obesity. However, eating habits and sedentary lifestyle are also two substantial factors in inducing overweight and obesity (1).

There is an obesity epidemic across the world (5). Hence, preventive measures are crucial to tackling the obesity epidemic and its related complications.

Epidemiologic information in the field of obesity is the basis for making health strategy decisions and planning health programs. High prevalence of obesity, overweight and their risk factors have reported among Iranian adults (6–8). To the best to our knowledge, there is no prior research in relation to the prevalence rates of obesity and overweight and the determination of associated risk factors in southwest of Iran.

The present study aimed at determining the prevalence rates of obesity, overweight, central obesity and the relevant risk factors in an urban population aged ≥20 in southwest of Iran. It was an attempt to demonstrate the status of this public health problem to help reduce it as much as possible.

## Materials and Methods

### Study design and population

This population-based cross-sectional study with a sample of 2575 subjects (1388 female and 1187 male) from an urban population aged ≥ 20 was performed using random cluster sampling method in 6 health centers in Ahvaz, center of Khuzestan Province (located in southwest of Iran), in 2015.

The study was approved by the Ethics Committee of Ahvaz Jundishapur University of Medical Sciences. After obtaining informed consent from volunteers, they were enrolled in the study.

A questionnaire consisting of age, sex, marital status, history of parental obesity, smoking, parity, ethnicity and educational level was filled for each person. Weight, height, body mass index (BMI), as well as abdominal and waist circumference were measured in each participant. The inclusion criteria were the age (≥20) and the participant’s belonging to the urban population. Pregnant women and homeless people were excluded.

### Definition of terms

Anthropometric indices were measured in subjects wearing light clothing without shoes. According to the standard protocol, waist circumference (WC) was measured at the midpoint between the lowest rib and the upper lateral border of the right iliac crest. Body mass index (BMI) was calculated by weight in kilogram divided by the square of height in meter (kg/m^2^).

The diagnosis of obesity was confirmed by the standard method proposed by WHO (9, 10). Accordingly, the BMI of 25–29.9 kg/m^2^ was considered overweight and that of ≥30 kg/m^2^ as obesity. Abdominal obesity was diagnosed according to WC with cutoff point of WC>88 cm for females and WC>102 cm for males.

### Statistical analysis

Descriptive statistics including frequency and percentage were used to present the categorical data.

To deal with the data analytically, logistic regression analysis was utilized to estimate the crude and age-adjusted odds ratio (OR) of obesity as well as the central obesity for different levels of baseline characteristics and lifestyle factors. The 95% confidence interval of OR was calculated as well. The *P*-value of less than 0.05 was regarded as the statistically significance level. SPSS software 16 (Chicago, IL, USA) was used for data analysis.

## Results

From 2575 participants, 1187 (47%) were men and 1388 (53.9%) were women. Their mean age was 41.07± 13.52 yr. The participants’ baseline characteristics are depicted in Table 1.

**Table 1:** Baseline characteristics of participants

***Variable***	(Male:1187)^1^^*^	(Female:1388)^*^	***(Total:2575)***

***Characteristics***	***N(%)***	***N(%)***	***N(%)***
**Age(yr)**			
20–29	273(23)	330(23.8)	603(23.4)
30–39	228(19.2)	368(26.5)	596(23.1)
40–49	278(23.4)	360(25.9)	638(24.7)
50–59	222(18.7)	206(14.8)	428(16.6)
60–69	119(10.0)	79(5.7)	198(7.6)
>=70	48(4.0)	25(1.8)	73(2.8)
**Education**			
Illiterate	118(9.9)	259(18.7)	377(14.6)
Primary level	210(17.7)	319(23.0)	529(20.5)
Elementary level	190(16.0)	207(14.9)	397(15.4)
High school and Diploma	406(34.2)	441(31.8)	847(32.8)
University level	263(22.2)	162(11.7)	425(16.5)
**Marital Status**			
Single	184(15.5)	161(11.6)	345(13.4)
Married	973(82.0)	1135(81.8)	2108(81.8)
Others(divorce and widow)	9(0.8)	71(5.1)	80(3.1)
**Parental Obesity**			
Absent	832(70.1)	932(67.1)	1764(68.5)
Present	340(28.6)	446(32.1)	786(30.5)
**Smoking Status**			
Smoker	208(17.5)	14(1)	222(8.6)
Non-smoker	970(81.7)	1366(98.4)	2336(90.7)
**Parity**			
Nulliparity	-	117(8.4)	-
Less than five	-	601(43.3)	-
Five or more	-	396(28.5)	-
**BMI(kg/m^2^)**			
<19	32(2.8)	41(2.8)	73(2.8)
19–24.99	425(36.5)	406(28.1)	831(31.9)
25–29.99	487(41.9)	527(36.4)	1014(38.9)
>30	219(18.8)	472(32.6)	691(26.5)
**Waist Circumference(cm)**			
>102	188(16.1)	-	-
<88	-	564(38.7)	-

* Missing data lead to lack of corresponding of sum of frequencies of subclasses with total sample size

The overall prevalence of obesity and overweight were 26.5% and 38.7%, respectively. The total prevalence rate of abdominal obesity was 28.6% (39.3% in women and 16% in men). Abdominal obesity was predominant in women compared to men (in a ratio of 3:1) (*P*<0.001).

The prevalence of obesity and abdominal obesity increased up to the age of 60 in both men and women and decreased thereafter except for the prevalence of abdominal obesity in women which increased without any lag. Considering the rates of obesity in all age groups, there was a consistent predominance of women to men.

The minimum rates of obesity in women and men were 16.46% and 13.19% in the age groups of 20–29 yr and the maximum rates were 47.29 and 22.17 in the age groups of 50–59 yr, respectively (Table 2). Logistic regression was used to calculate the odds ratio of obesity to age-adjustment. With increasing the age, odds of obesity become higher until the age of 60 in both women and men and decrease thereafter (Table 3). The riskiest age group for obesity was 50–59 since their estimated OR obesity was 2.53 (1.79, 3.54).

**Table 2:** Classification of weight according to BMI, sex and age groups^2^^*^

***BMI***	***Underweight***	***Normal***	***Overweight***	***Obese***	***Abdominal obesity***

	***(BMI‹19)***	***(19≤BMI≤24.9)***	***(25≤BMI≤29.99)***	***(BMI≥30)***	
**Age Groups(yr)**					
**Male**					
20–29	22(8.06)	128(46.89)	87(31.87)	36(13.19)	19(6.96)
30–39	5(2.2)	91(40.0)	86(37.89)	45(19.82)	42(18.42)
40–49	3(1.09)	81(29.45)	131(47.64)	60(21.82)	46(16.55)
50–59	1(0.45)	74(33.48)	97(43.89)	49(22.17)	49(22.07)
60–70	1(0.60)	51(30.72)	85(51.20)	29(17.47)	32(19.16)
**Female**					
20–29	25(7.62)	162(49.39)	87(26.52)	54(16.46)	46(13.94)
30–39	5(1.36)	87(23.64)	149(40.49)	127(34.51)	143(38.86)
40–49	1(0.27)	67(18.66)	155(43.18)	146(37.88)	168(47)
50–59	4(1.97)	39(19.21)	64(31.53)	96(47.29)	120(58.25)
60–70	2(1.96)	28(27.45)	35(34.31)	37(36.27)	64(61.54)

* Data is expressed in N (%)

Female gender could be regarded as a potential risk factor in obesity since age-adjusted estimates of OR for women were approximately 3 times greater than those of men (*P*<0.001) (Table 3).

**Table 3:** Logistic regression analysis of obesity and abdominal obesity risk factors

***Variables***	***Crude OR (95%CI) Of obesity***	***Age-adjusted OR (95%CI) Of obesity***	***Crude OR (95%CI) of abdominal obesity***
**Age groups (yr)**			
20–29	(Ref)	-	(Ref)
30–39	1.90(1.36,2.54)	-	3.93(2.75,5.60)
40–49	2.09(1.52,2.86)	-	5.73(3.91,8.40)
50–59	2.52(1.79,3.54)	-	5.32(3.34,8.46)
60–70	1.78(1.15,2.75)		4.75(2.48,9.10)
**Education**			
Illiterate	2(1.25,2.60)	1.73(1.19,2.51)	1.89(0.59,5.99)
Primary level	2.24(1.55,2.76)	2.14(1.52,3.00)	1.78(0.57,5.60)
Elementary level	1.93(1.35,2.69)	1.95(1.37,2.78)	1.37(0.43,4.32)
High school	1.21(0.89,1.70)	1.27(0.92,10.75)	1.42(0.43,4.67)
University level	Ref	-------	(Ref)
**Marital Status**			
Single	(Ref)	-----	(Ref)
Married	3.9(2.13,4.56)	2.4(1.75,2.99)	1.40(0.94,2.10)
Others(divorce and widow)	2(0.73,2.60)	2.2(0.84,2.88)	1.86(0.98,3.55)
**Parental obesity**			
Absent	(Ref)	-----	(Ref)
Present	2.45(1.60,2.90)	2.7(1.71,3.2)	2.92(2.37,3.59)
**Smoking status**			
Smoker	0.69(0.43,1.02)	0.71(0.48,1.05)	0.76(0.50,1.15)
Non-smoker	(Ref)	-----	(Ref)
**Parity**			
Nulliparity	(Ref)	-----	-----
Less than five	5.33(2.9–6)	2.5(1.18–3.12)	-----
Five or more	7(4.01,12.23)	4.16(2.17,7.65)	-----
**Gender**			
Male	(Ref)	------	(Ref)
Female	1.93(1.56–2.38)	2.99(1.86–3.21)	3.39(2.70,4.25)

Considering the level of education, nearly half of the study subjects had high school or higher levels of education. Only 15% of the subjects were illiterate. The lower the level of education, the higher the risk of obesity was.

Women who had at least one parity were at risk for obesity and the risk increased with a greater number of parties: women with five or more parties would be at the risk of obesity four times greater than nulliparous women (*P*<0.001). Having a positive family history of obesity was evident in approximately one–third of the study subjects. Smoking did not increase the risk of obesity (Adjusted OR=0.71 (0.48–1.05) (*P*=0.089).

Baseline characteristics such as higher age, female gender, parenteral obesity, educational status, and marital status increased the risk of abdominal obesity (*P*=0.0001). Smoking did not show any significant relationship with abdominal obesity (*P*>0.64). After doing the logistic regression, only the age, female gender, and parenteral obesity were significantly relevant to abdominal obesity (Table 3). A comparison between the prevalence of obesity and overweight in different countries is presented in Table 4.

**Table 4:** Comparison between prevalence of obesity and overweight in different countries

***Country***	***Prevalence of obesity (%)***	***Prevalence of overweight (%)***
**Iran**					
Present study	26.5	38.7
North(14)^*^	30.5	37.8
Center-to-east(16)	26.3	40.6
North(13)	25.19	12.2
**Other countries**					
United States(26, 30)	21.2	36
Bahrain(18)	36	33
Kuwait(18)	47.48	32.9
Lebanon(18)	15	35.93
Libya(18)	6.45	20.15
Morocco(18)	11.74	30.84
Oman(18)	19.09	28.79
Saudi Arabia(18)	41	36

* Numbers in country columns are reference numbers

## Discussion

The findings of our study show an overall prevalence rate of 26.5% for obesity and 38.7% for overweight (65.2% for both) in Iranian population aged >=20 yr living in Southern Iran.

Generally, 69.1% of women and 60.6% of men were found to be overweight or obese in this study. In Thailand, 22.4% of men and 34.3% of women had general obesity. The percentage was higher in women than men but their study reported a lower prevalence in comparison with the present study (11). In south of Tehran, the obesity rates of 29.1% for women and 14.2% for men have been reported (12). To some extent, it was similar to our findings.

In two other studies in of the North of Iran, Gilan, and Mazandaran, the overall prevalence rate of obesity and overweight was higher than that of our study (13, 14); it may be due to lower level of physical activity in the population of Ahvaz than the population of the above-mentioned cities where people usually do agricultural activities. A comparison between the prevalence rates of obesity and overweight in different countries is shown in Table 4.

In another study in district 17 of Tehran, close to the center of the city, the prevalence rates of obesity and overweight were similar to ours. In the latter study, the prevalence rates of obesity and overweight were 30.5% and 37.8%, respectively (15).

In one of the Eastern provinces of Iran, obesity and overweight rates were reported at 26.3 and 40.6, respectively. Their results were in agreement with ours (16) (Table 4). In those studies conducted in Northern part of Iran, the prevalence rates of obesity and overweight were less than ours and that of the two previously mentioned studies (13–16).

The higher rate of obesity in southwest and central parts of the country may be explained by lifestyle factors including eating habits (such as high-calorie diets and fast foods which seems to be more common in main cities than the agricultural areas in Northern provinces). Moreover, the physical activity level seems to be much less in main cities than the agricultural towns. Furthermore, jobs with severe physical activity are more frequent in villages and agricultural areas which are more abundant in northern provinces than the central parts of the country.

The prevalence of obesity varies substantially between countries. The prevalence rate of obesity was reported as low as ≤5% in Japan, China, and some regions in Africa up to as high as ≥75% in urban Samoa (17). Obesity is frequent in countries with a high economic status like the US, New Zealand and Australia (17). In the Eastern Mediterranean Region (EMR) countries, the prevalence rate was 6.5% to 47.48% (Table 4).

There are several factors associated with the occurrence of obesity but energy intake and inactivity are among the most convincing factors. Economic development during the last 50 years in most of the EMR countries has resulted in changes in dietary habits. The most notable ones are the rise in fat consumption, especially that of saturated fats, cholesterol, and refined carbohydrates and a decrease in consuming poly-unsaturated fatty acids and dietary fibers. This nutrition transition together with a sedentary lifestyle and increased level of stress has resulted in a steeple rise in the prevalence of obesity and other non-communicable diseases (18). Hence, there is a strong association between lifestyle and obesity. Several studies from different countries have confirmed this hypothesis (14, 19, 20).

The results of the present study showed the negative association between education and obesity, also observed in other studies (14, 21, 22).

The higher prevalence rate of obesity in women in the present study may be explained by some factors: In Iranian female population, the age of marriage is usually less than that of men. On the other hand, marriage is a potential risk factor for obesity (OR=2.4 CI (1.75–2.99)). Therefore, higher marriage rate at younger age may contribute to female obesity. Metabolic syndrome had high prevalence in our population and its prevalence increased in line with increasing the age and the BMI. Women were at higher risk for metabolic syndrome than men and sedentary lifestyle was the reason for this higher prevalence of obesity and abdominal obesity in women (23).

The results of the present study showed that with growing the number of parties more than five, the odds of obesity becomes doubled (from 2.5 to 4.16). The positive association between obesity and parity was also observed in other studies. However, such a strong association was not seen in similar studies(14, 24).

In the present study, there was no association between parity and central obesity after OR adjustment. Crude odds ratio showed a strong association between parity and anthropometric markers of obesity but after the adjustment, the association remained only for BMI (general obesity) (25). Therefore, parity can increase adiposity but not necessarily through a central pattern. The results of our study, in consistence with other studies, confirm an epidemic of overweight and obesity in both developing and developed counties. From 1986 to 2000, the prevalence rate of obesity among Americans has increased from 1 in 10 to 1 in 5 subjects (about two times) (26). Nevertheless, obesity did not show any significant increase among American women overall. For men, however, there was a significant linear trend over the past 12 yr (from 1999 to 2010) (27).

There is a trend for increasing the obesity prevalence rate in developed and developing countries. The obesity rate in southwest of Iran is somewhat lower than what is reported for the surrounding countries. Differences in the lifestyle pattern, social culture, economic status, physical activities, nutritional status and the selection of different cut–off points for defining obesity may explain the different prevalence rates of obesity in different parts of the world.

The observed significant increase in the prevalence rate of obesity in line with growing the age, in present study, was reported by all other studies (14, 28). This phenomenon was seen in most EMR countries up to 60 yr of age when obesity showed a decline (18).

By considering the results derived from logistic regression analysis, risk factors for abdominal obesity and general obesity are similar. Increasing age, presence of parental obesity, marriage and low level of education are the chief risk factors for both obesity and abdominal obesity in women and men in south-west of Iran.

There are some limitations in our study including the nutritional and occupational limitations. Moreover, data regarding leisure time and physical activities were not available for all participants.

There is an urgent need for public health prevention strategies to help modify health behaviors so as to decrease obesity and its subsequent complications such as diabetes, hypertension, coronary artery disease, and the like in the population of south-west Iran. An increase in physical activities is exerted not only in leisure time but in work time by walking or bicycle riding, sports facilities, lifestyle changes and replacing high fiber and low-fat diets for routine diets.

## Conclusion

Obesity and overweight have a high prevalence in south-west of Iran. This prevalence increases with increasing age. Several risk factors such as low level of education, parity, female gender, marriage, and familial history of obesity are associated with obesity.

## Ethical considerations

Ethical issues (Including plagiarism, informed consent, misconduct, data fabrication and/or falsification, double publication and/or submission, redundancy, etc.) have been completely observed by the authors.
